# Community-Level Urban Green Space Equity Evaluation Based on Spatial Design Network Analysis (sDNA): A Case Study of Central Wuhan, China

**DOI:** 10.3390/ijerph181910174

**Published:** 2021-09-28

**Authors:** Qing Li, Kaili Peng, Peng Cheng

**Affiliations:** 1 College of Public Administration, Huazhong Agricultural University, Wuhan 430070, China; qingL@webmail.hzau.edu.cn (Q.L.); chengfapeng@webmail.hzau.edu.cn (P.C.); 2 College of Humanities & Social Sciences, Huazhong Agricultural University, Wuhan 430070, China

**Keywords:** urban green space, equity, Gini coefficient, spatial design network analysis (sDNA), Wuhan

## Abstract

Urban green spaces (UGSs) provide numerous irreplaceable environmental and social benefits to humankind, but the lack of baseline information makes it difficult to propose a reasonable greening strategy so as to achieve an equitable allocation of community green spaces. This paper divides UGSs into three classes using the spatial design network analysis (sDNA) and quantifies the UGS accessibility of communities in central Wuhan. Based on these results and the Gini coefficient, we analyze the UGS equity of the spatial distribution at the community level, then propose future greening strategies both at the city and community levels. The results show that the railway station and old Wuhan city are the core areas of traffic network strength (TNS). UGSs are evenly distributed in the core areas of TNS, but the number of UGSs in non-core areas is small, and their distribution is relatively uneven, and the number of communities with medium UGS accessibility is the largest, carrying the densest residential population. Most communities perform well in terms of UGS equity, but the UGS equity of 163 communities, covering a population of more than one million, remains to be improved. The method and conclusions of this study will contribute to the future greening policy making of 965 communities in central Wuhan, thus promoting the orderly planning and high-quality construction of community living circles.

## 1. Introduction

A series of environmental issues and social injustices caused by rapid urbanization have become a common phenomenon worldwide [1,2]. In this context, urban green spaces (UGSs) with environmental, economic, and social benefits have received increasing attention [3,4,5]. The environmental benefits of UGS mainly include improving air quality [6,7], recharging groundwater [8,9], reducing urban noise pollution [10,11], and resisting urban flooding [12]. UGS can also maintain urban biodiversity by providing living space for animals [13,14,15]. In terms of economic benefits, the location and area of UGSs have important influences on land prices and property prices. In addition, UGS with landscape aesthetic characteristics can promote tourism and increase the economic income of local residents [16]. The social benefits of UGS are mainly manifested in improving the physical and mental health of residents and increasing their sense of well-being [17]. For example, there is clear evidence that the degree of urban greenery and forest area are important factors influencing residents’ psychological health [18], and that residents living in green environments tend to be more positive and mentally healthy [19]. Residents usually choose to visit parks with UGSs for fun and recreation during their leisure time to relieve stress and improve family relationship [20]. Although a large number of studies have confirmed that UGS plays an important role in achieving sustainable urban development and increasing the well-being of urban residents. With the increasing global urbanization and the huge differences in regional economic development levels, the existing UGSs can no longer meet people’s needs, especially for those developing countries experiencing explosive urban population growth. Therefore, it is urgent for relevant government departments to adopt effective policies to promote UGS equity.

The existing studies of green space equity are mainly conducted from the following three perspectives: geographical [21,22,23], social [24,25,26], and political [27,28,29]. Among them, the geographic perspective can objectively quantify the spatial equity of green spaces based on geographical information system (GIS) technology, and geographic perspective study introduces urban green space equity into the category of urban planning and management, which can undoubtedly provide an efficient and consistent policy framework for promoting a regional coordinated supply of UGS. Accessibility is an important concept in the study of green space equity from a geographical perspective. The current measurement methods of UGS accessibility mainly include the two-step floating catchment area (2SFCA) [30], buffer zone analysis [31], and network analysis [32]. The 2SFCA is used to calculate the accessibility of green space in two steps based on the supply and demand area [33]. This method is convenient, and it overcomes the limitation of administrative boundaries, but it ignores the actual resistance caused by the traffic road network. The buffer zone analysis is used to determine the scope in which a green space provides services by creating a buffer zone with a certain distance from the green space and its neighboring lands [34]. This method can intuitively reflect the overall level of green space coverage, but it tends to ignore the influence of actual resistance factors such as traffic road networks, thus resulting in the deviation of research results from the actual situation. The above literature review reveals that although there are various methods for evaluating the accessibility of green spaces, these methods are often limited by a certain factor, thus differing from the actual situation. In recent years, the spatial syntax has been gradually applied to accessibility analysis [35,36]. The spatial syntax is a new technique for studying the internal morphology and inherent social logic of cities, and this technique relies on detailed urban maps for modelling based on the spatial relationship and the road topological relationship, thus it provides an accurate analysis of spatial use potential from both global and local perspectives [37,38]. Therefore, we can adopt the spatial syntax to measure green space accessibility and evaluate green space equity accurately and objectively from a geographical perspective.

Macroscopically, the existing studies have mainly focused on an individual city [39,40] or urban agglomerations [41,42], and there is a lack of relevant research at the microscopic level [43,44]. A microscopic evaluation on green space equity will contribute to the precise implementation of urban greening policies. The community is the basic unit of urban governance and the smallest living unit where urban residents can enjoy a wide range of urban services [45]. Some international cities rich in urban planning experiences have successively put forward the concept of community living circles [46,47]. China’s metropolises such as Shanghai [45], Beijing [48], and Guangzhou [49] also have a practical basis in the construction of community living circles. In 2021, China’s Ministry of Natural Resources officially released the Technical Guideline for Community Living Circle Planning, which aims to create healthy, safe, green, and vibrant community living circles [50]. UGS, as an important part of community infrastructure, is crucial for the coordinated development of communities within cities and the enhancement of residents’ well-being. An in-depth analysis of the differences in green space equity among different communities within cities and the proposal of rational urban green space planning and policy recommendations are important for achieving the high-quality and sustainable development of community living circles.

It should be noted that human activities and demands are at the heart of community green space infrastructure development. One study in Seoul found that green spaces present an equitable allocation when spatial location matches population distribution [51]. Many national and international organizations commonly use census data to evaluate green space equity at the city level [52,53], and population is also an important factor in green space policy making in some developed cities [54]. For example, the government of Berlin, Germany, mandates 6 m^2^ of green space per person within the coverage of a community park [55]. The Dutch government entitles 60 m^2^ of green space for residents within 500 m of a settlement [56]. The integration of population data with urban green space information will contribute to improving the accuracy and effectiveness of green space equity evaluation [57,58]. For these reasons, population size is an important factor to be considered when the equity of green space is evaluated at the community level.

Despite the current extensive discussions on UGS equity, several aspects have not been well addressed. Compared with the existing literature, the major contributions of this study are as follows. Firstly, most previous studies on measuring UGS accessibility based on spatial syntax theory [59,60,61] have mainly used software such as Depthmap, which has certain limitations in the operation process. The spatial design network analysis (sDNA) model based on spatial syntax is highly compatible with the geographic information system (GIS), and it has the advantages of operational stability and indicator comprehensiveness for large-scale data analysis. Secondly, most previous studies of green space equity have focused on the green space at the spatial level and on the relationship between the UGS demand and supply. Few studies have investigated the relationship between UGS and the spatial distribution of population. Finally, the number of UGS equity studies at the typical community level is negligible. 

Against this background, this study focused on evaluating the accessibility and spatial equity of UGSs based on spatial syntax through a case study of central Wuhan. Specifically, this study is aimed to (1) analyze the current status of the traffic road network and classify green spaces in the central Wuhan based on the sDNA model; (2) construct a community-level green space accessibility and equity measurement model; (3) quantify the green space accessibility and equity of each community and reveal the relationship between population size and green space equity in each community; and (4) propose future greening policy recommendations for central Wuhan, referring to the results of the above analysis. Our findings will help rapidly developing cities to improve green space planning and construction at the community level. In addition, this spatial analysis approach applies to examining green space services in cities with different transportation network types to inform regional planning and help researchers understand how to improve the social equity of public green space.

## 2. Materials and Methods

### 2.1. Research Framework

**Figure 1:**
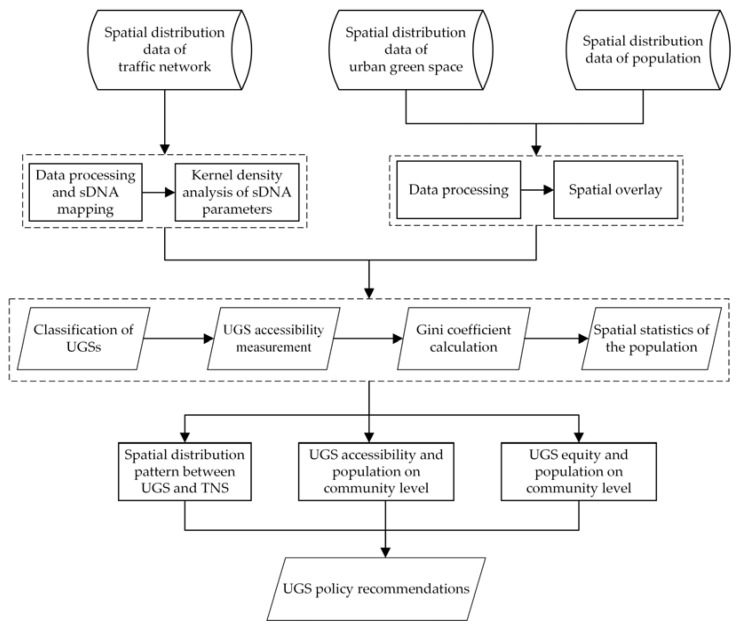
Research framework.

Based on the research background, this research framework design is shown in Figure 1. First, we perform data processing and modeling analysis based on the traffic network data and then analyze the current status of the traffic network based on the results of the relevant parameters of the sDNA model. Additionally, we classify green spaces in central Wuhan. In the next step, we measure the community-scale UGS accessibility and equity based on the classification of UGSs and the Gini coefficient measurement formula. Finally, based on the measurement results, we propose a future greening strategy for central Wuhan, taking into account the spatial distribution of the population and the current situation of the traffic road network.

### 2.2. Study Area

**Figure 2:**
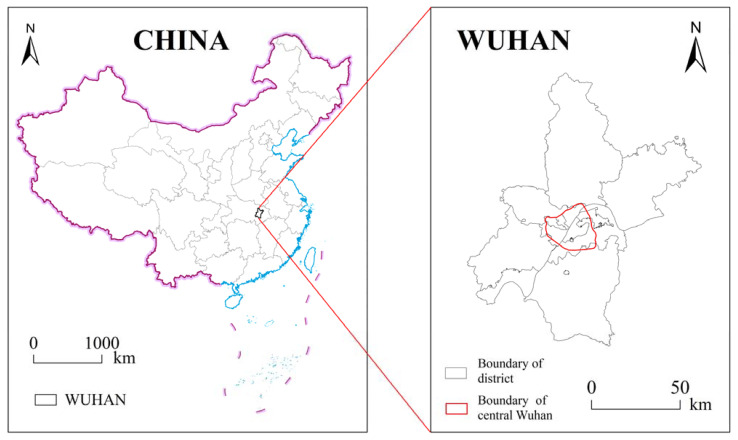
Location of central Wuhan in China.

Wuhan is located in the eastern part of Hubei Province (29°58′–31°22′ N, 113°41′–115°05′ E), and it is the core city of the Yangtze River Economic Belt and Wuhan Metropolitan Area. There is a dense road network consisting of three rings of traffic routes distributed from inside to outside. The Wuhan City Territorial Spatial Master Plan (2017–2035) takes the areas within the Third Ring Road as central Wuhan, which is both the core area of Wuhan’s transportation services and the main activity area of the city’s residents [62]. Central Wuhan covers a total of 965 communities belonging to 11 districts in Wuhan city, including Jiangan, Jianghan, Qiaokou, Hanyang, Wuchang, Hongshan, Caidian, Dongxihu, Huangpi, Qingshan, and Jiangxia districts, with a total land area of 525.52 km^2^, accounting for 6.13% of the city’s total area (Figure 2). Central Wuhan was selected as the study area for the following reasons. Firstly, most previous studies on the equity of UGS in specific cities in China have focused on the eastern coastal zone [63,64], which has advantages in terms of geographic location and economic development. In recent years, Wuhan, Zhengzhou, and other cities in central China have been developing rapidly. Thus, UGS studies in these regions will enrich existing research findings. Secondly, Wuhan is experiencing rapid population and economic growth. In 2020, Wuhan has a resident population of 11.21 million, accounting for 19.16% of the province, and a GDP of RMB 1561.66 billion, accounting for 35.95% of the province. Central Wuhan is the core place of the city’s population and economy, and it is crucial for improving social equity to provide good green space services for the huge number of residents while developing the economy. Finally, Wuhan city government has introduced a series of UGS planning policies to improve the uneven distribution of green spaces. For example, the Wuhan city government plans to build 6.5 million m^2^ of green space within the main urban area [65]. Therefore, achieving an equitable distribution of green space resources in central Wuhan has become an important goal for the local government.

### 2.3. Data Collection and Processing

**Figure 3:**
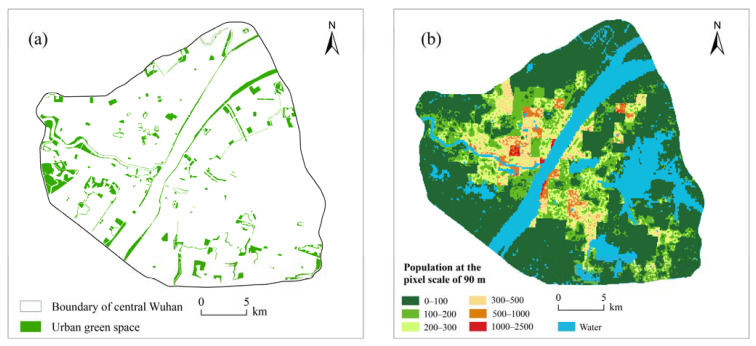
Urban green space information (**a**) and population information (**b**) in central Wuhan.

In this study, we collected data including administrative division data, traffic road network data, green space data, and population distribution data in the study area (Figure 3). The Wuhan administrative division data were obtained from the Resource and Environment Science and Data Center of the Chinese Academy of Sciences (https://www.resdc.cn/, accessed on 20 April 2021), and the road network data and public green space data of central Wuhan were obtained from Gaode Map (https://www.amap.com/, accessed on 24 April 2021). In this study, public green spaces are defined according to World Health Organization standards, which state that each urban green spaces should be greater than 1 hm^2^ [37]. We obtained 306 public green spaces in central Wuhan with a total area of 42.70 km^2^. These UGSs mainly consist of woodlands, grasslands, and wetlands, which have the functions of parks and attractions. In addition, we downloaded population distribution data from WorldPop (https://www.worldpop.org/, accessed on 6 April 2021) covering the study area with a spatial resolution of approximately 90 m. All data were converted to the same coordinate system and stored in the same geographical database.

### 2.4. Spatial Design Network Analysis

Spatial design network analysis (sDNA), as an emerging software for spatial syntax research, improves the analysis capability of spatial syntax on region modeling. Although its advantages have been acknowledged, this model has not been used widely in the field of UGS research since its development. Therefore, this paper quantitatively evaluates the accessibility and equity of UGS using the sDNA model with excellent compatibility with GIS. Several parameters calculate the traffic network morphology in the spatial syntax. The sDNA-related parameters used in this study include closeness and betweenness, similar to the accessibility and traffic potential of telecommunication networks proposed by Shimbel [66] and Freeman [67]. The closeness (NQPDA(*x*)) measures accessibility, which is usually the average reverse distance from a given link or node on the network to all other links or nodes within a local radius, and it measures the average difficulty of getting from each link to all possible destinations of radius *x* [68], and a high closeness means that there is high accessibility and centrality of a certain lot, and it is calculated according to Equation (1). The betweenness (TPB_t_(*x*)) refers to the maximum travel distance determined by the radius, assuming that a given network is populated with entities from everywhere to everywhere else. A high betweenness means there is a strong road network passability. The betweenness usually reflects the traffic development potential of a region, and it is calculated according to Equation (2). In this paper, we use an sDNA model to calculate the parameters of closeness and betweenness in central Wuhan. To analyze the local and global characteristics of the road network according to the travel means and time costs of the residents in the study area, 1.2 km and N (infinity) are selected as the radii, respectively.

(1) $$NQPDA(x)=\sum_{y\in Rx}\frac{p(y)}{d(x,y)}$$

where *p*(*y*) is the weight of node *y* within the search radius R. In continuous spatial analysis, *p*(*y*)∈ [0, 1], and in discrete spatial analysis, *p*(*y*) has a value of 0 or 1. *d*(*x*, *y*) is the shortest topological distance from node *x* to node *y*.

(2) OD(y,z,x)={1,x lies on the shortest path from y to z12,x≡y≡z12,x≡y≡z13,x≡y≡z0,other situationsTPBt(x)=∑y∈N∑z∈RyOD(y,z,x)P(z)Links(y)

where *OD* (*y*, *z*, *x*) is the shortest topological path between nodes *y* and *z* through node *x* in search radius R; Links(*y*) is the total number of nodes in search radius R for each node *y*; and *P*(*z*) is the weight of node *z*.

### 2.5. Kernel Density Analysis

Kernel density is estimated with each sample point as the search center by using a mathematical function varying from 1 to 0 corresponding from the center to the boundary to form a smooth curved surface that is suitable for each sample point. Kernel density analysis can transform linear road network morphological data into surface data for more intuitive hotspot analysis, and it is calculated using Equation (3). In this paper, based on the kernel density estimation tool, the parameter data are calculated using sDNA with 90 m as the image size and 2000 m as the search radius, and are transformed from linear to faceted for subsequent analysis. We superimposed the kernel density analysis results of closeness and betweenness at radius of 1.2 km and N with the same weights, and the superimposed results were defined as traffic network strength (TNS) at local scale and city scale, respectively.

(3) $$f_{n}\left(x\right)=\frac{1}{nh}\sum_{i=1}^{n}k\left(\frac{x-x_{i}}{h}\right)$$

where $k(\frac{{x-x}_{i}}{h})$ is the kernel function; *h* is the search radius, and *n* is the number of known points within the search radius, namely, the number of sample points. The calculation result is the kernel density at point *x*.

### 2.6. Community-Based UGS Accessibility Measurement

**Figure 4:**
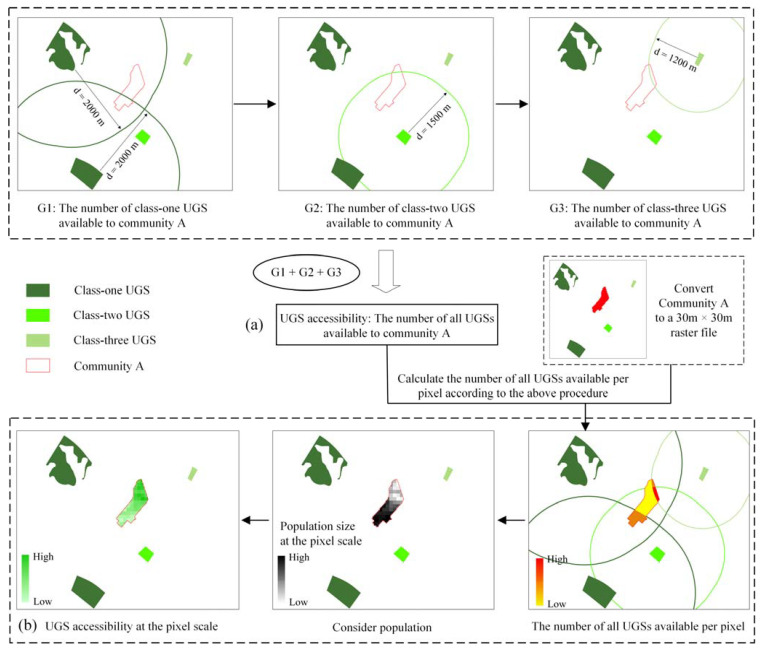
Quantification of community-scale UGS accessibility (**a**) and quantification of pixel-scale UGS accessibility (**b**).

In order to objectively and accurately reflect the accessibility of UGS at the community level, we design an algorithm incorporating residents’ travel costs and traffic intensity with reference to their travel means and time costs (Figure 4a). UGS accessibility at community scale is quantified as follows. (1) According to Jenks natural break approach, traffic network strength is divided into six categories from large to small, and the first three categories are the core of TNS. The UGS covered by the core of TNS at the city scale is defined as the first class of UGS in this paper, and the UGS covered by the core of TNS at the local scale is the second class of UGS. All other UGSs are considered as the third class, and the highest class is taken when a certain UGS is covered by more than one class. (2) Most studies have found that 15 min is the optimal walking time for residents to visit UGSs. We measure accessibility based on the number of UGSs that residents in the community can reach within 15 min. The higher the TNS value is, the wider the UGSs that residents in the community can enjoy at the same time are. Due to the difference in TNS around each class of UGS, residents within each community can reach class one UGSs, class two UGSs, and class three UGSs within 2, 1.5, and 1.2 km in 15 min, respectively. (3) The community-scale UGS accessibility is defined as the number of all UGSs that can be reached by the community residents within 15 min in a set scenario.

### 2.7. Gini Coefficient-Based UGS Equity Measurement at Community Scale

The Gini coefficient is originally used as an economic indicator to measure the income disparity between residents in a region or country [69], and its value is between 0 and 1; when it is close to 0, the distribution tends to be more equal [70]. In recent years, the Gini coefficient has also been gradually applied to evaluate the equity of the spatial distribution of education [71], health [72], and UGS [42,73]. According to the connotation of environmental justice [74], the equity of UGSs in this study is defined as the right of every resident to have equal access to green spaces regardless of the environment of their residence and the local natural endowment. In this study, UGS equity is defined as the variability in the amount of residents’ access to green space in a community within 15 min at the pixel scale, as shown in Equation (4) [75]. First, we converted the community vector surface data into raster data with a pixel of 30 m to calculate the number of each class of UGSs that residents can reach in 15 min at the pixel scale (Figure 4b). Second, we took population data into account, and the accessibility at the pixel scale was calculated as the number of UGSs at the pixel scale divided by the number of people covered by the pixel. Finally, the pixel-scale UGS accessibility is used to calculate the Gini coefficient at the community level.

(4) $$G_{comm}=1+\left(\frac{1}{n}\right)-\left(\frac{2}{\bar{M}\cdot n^{2}}\right)\sum_{i=1}^{n}\left[\left(n-i+1\right)\cdot M_{i}\right]$$

where *G_comm_* represents the inequity of access to UGS services for residents within the community; *n* is the number of pixels within the community boundary; *M_i_* represents the pixel-scale UGS accessibility of the ith pixel; and $\bar{M}$ is the average of the pixel-scale UGS accessibilities within the community.

## 3. Results

### 3.1. Kernel Density Analysis of sDNA Model

**Figure 5:**
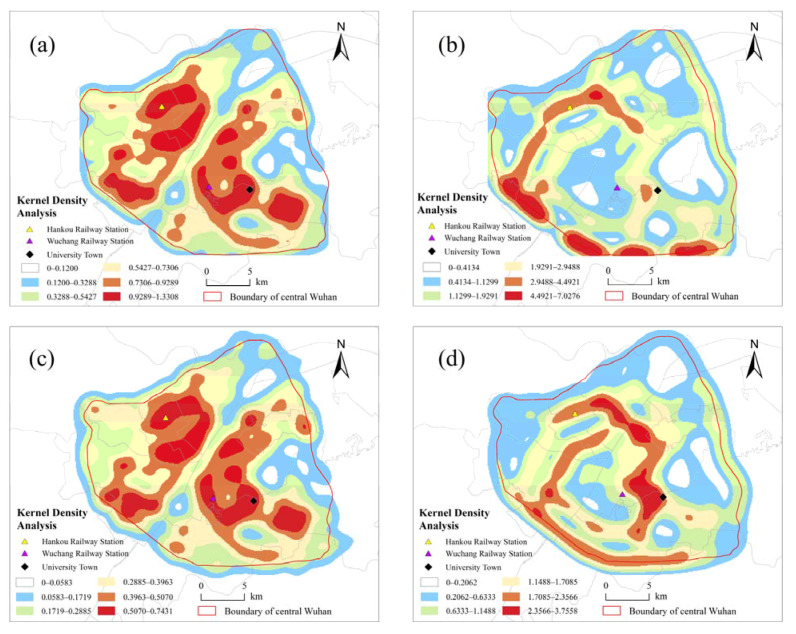
Kernel density analysis of closeness (**a**) and betweenness (**b**) at the city scale (R = N), and kernel density analysis of the closeness (**c**) and betweenness (**d**) at the local scale (R = 1.2 km).

The results of the kernel density analysis of closeness and betweenness are divided into six categories according to the Jenks natural break classification (Figure 5). The results of the kernel density analysis of closeness at the city scale (R = N) show that the closeness of central Wuhan exhibits the characteristic of spreading from a certain central point to the surrounding areas, and that there are significant differences in the closeness among various areas. The closeness of the areas around the Third Ring Road is relatively low, whereas the closeness of the areas near transportation hubs (such as railway stations) and tourist attractions is higher. At the local scale (R = 1.2 km), the areas with good closeness are still concentrated in the above-mentioned areas, and the coverage of areas with medium closeness has been expanded.

The results of the kernel density analysis of the betweenness reveal the ring-like characteristics of the traffic network in central Wuhan. The betweenness at the city scale (R = N) still varies significantly spatially. The southern part of the third ring road exhibits the highest traffic development potential, while the northern part displays poor traffic development potential. The traffic development potential is strongest in the areas around Hankou Railway Station and Wuchang Railway Station within the Second Ring Road. At the local scale (R = 1.2 km), the traffic road network is well developed in the first and second ring roads, and only parts of the third ring road area show good development potential. The spatial difference in this parameter reflects the characteristics of traffic ring development in Wuhan, which is consistent with the actual situation.

### 3.2. Classification of UGS

**Figure 6:**
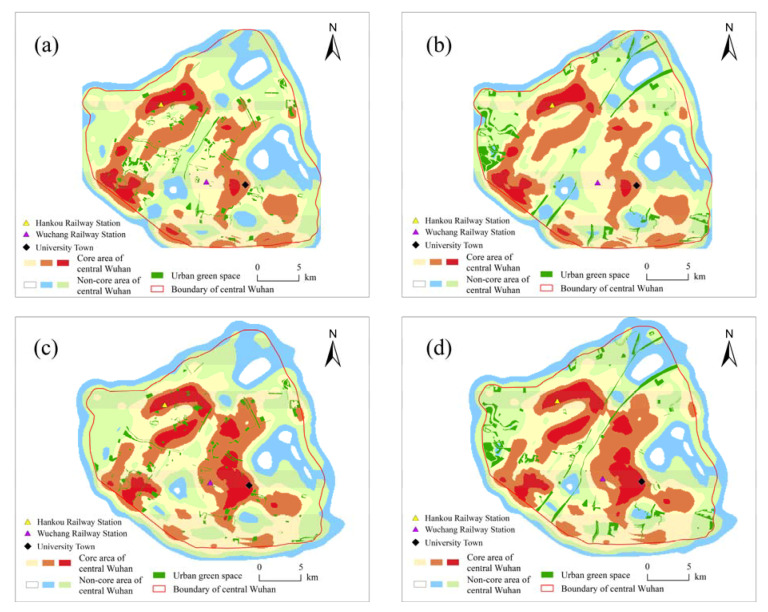
Urban green space in core area (**a**) and non-core area (**b**) at the city scale (R = N), and urban green space in core area (**c**) and non-core area (**d**) at the local scale (R = 1.2 km).

A road network with high closeness is more attractive to regional travel traffic flows, and high betweenness represents a strong passibility of the road network. We superimposed the results of the kernel density analysis of closeness and betweenness with the same weights, and the superimposed results were defined as traffic network strength (TNS). According to the classification of Jenks’ natural fracture method, TNS is classified into six categories, and the first three categories are the core of the central Wuhan transportation network (Figure 6). The classification of transportation networks shows that the core scope of the study area at the city scale (Figure 6a,b) was roughly in line with that at the local scale but with a reduced TNS. At the local scale, the old Wuhan city is the core of the transportation network, and Wuhan railway station and the university town areas in Hongshan District are subcore areas (Figure 6c,d). At the city scale, the number of UGSs in the core and non-core areas is 188 and 118, respectively. At the local scale, the number of UGSs in the core and non-core areas is 203 and 103, respectively (Table 1). The UGSs in the core areas at both scales are evenly distributed and well connected, although the coverage of UGSs in the core areas at the local scale is larger than that at the city scale (Figure 6a,c), which might be attributed to the fact that the local scale better reflects the perspective of the residents, and that some UGSs close to the city center and that are easily accessible have stronger appeal. In the non-core areas (Figure 6b,d), UGSs are relatively scattered and poorly connected to each other due to the weak transportation network attached to these UGSs.

**Figure 7:**
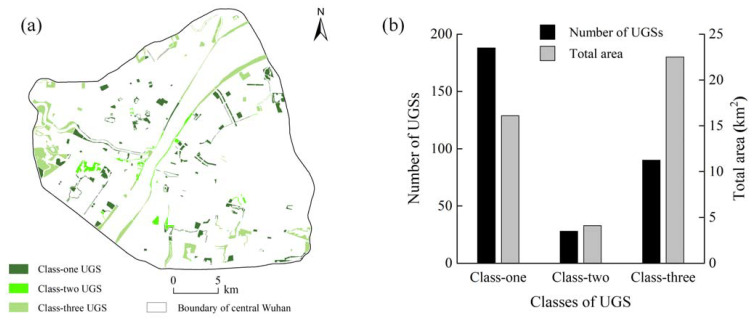
Spatial distribution of different classes of UGSs (**a**) and statistical results of the number and area of different classes of UGSs (**b**).

In terms of the current distribution of TNS in core and non-core areas at different scales (R = 1.2 km and R = N), the UGSs in the central Wuhan were classified into three categories (Figure 7a). We further calculated the number and area of different classes of UGS (Figure 7b). The results show that the first class of UGS is more evenly distributed throughout central Wuhan with the highest percentage among the three classes of UGSs (as high as 188). Although the number of the third class of UGS is less than half of that of the first class of UGS (the number is 90), its area is the largest among the three classes (as high as 22.49 km^2^). The second class of UGS is mostly concentrated in the southwest part of central Wuhan, with the smallest number and area among the three classes.

### 3.3. Accessibility of UGS at the Community Level

**Figure 8:**
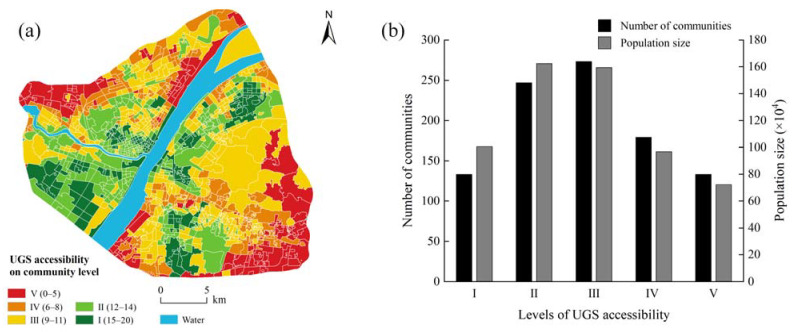
UGS accessibility level of communities (**a**) and statistical analysis of number and population of communities (**b**).

Based on the results of TNS distribution and green space classification in central Wuhan, we measured the UGS accessibility at the community level using the algorithm described in Section 2.5 (Figure 8a). The UGS accessibility is classified into five levels based on the number of UGS that can be reached within a certain range. The communities with high accessibility are mostly concentrated in the central and southwest corners of the study area, and their UGS distribution is relatively concentrated. Most poorly-accessible communities whose UGS accessibility is mostly rated as level III are located in the southeastern part of the study area, and these communities have small area. The number and population distribution of the communities at different UGS accessibility levels were further analyzed (Figure 8b). The results show that the number of communities at UGS accessibility level II and level III is the largest, and these communities have the densest residential population. Only 133 communities covering 721,200 people have the worst UGS accessibility, indicating that the majority of residents in these communities have little access to adequate and high-quality UGS services. In contrast, the 133 communities with the best accessibility cover a population of 1,004,700 with more than 15 UGSs. This indicates that the current UGS in central Wuhan is still unable to meet the demands of a large number of residents. Therefore, it is urgent to propose an objective and reasonable green space planning. In addition, we analyzed the distribution of communities with different accessibility in core areas (R = 1.2 km) and non-core areas (R = N). The results show that the communities with a good accessibility (level I and level II) are primarily distributed in the core areas (total area = 122.61 km^2^), and a few are distributed in non-core areas (Total area = 42.40 km^2^). The communities with poor accessibility (level IV and level V) have a distribution area of 90.43 km^2^ in the core area and 68.55 km^2^ in the non-core area.

### 3.4. UGS Equity Assessment at the Community Level

**Figure 9:**
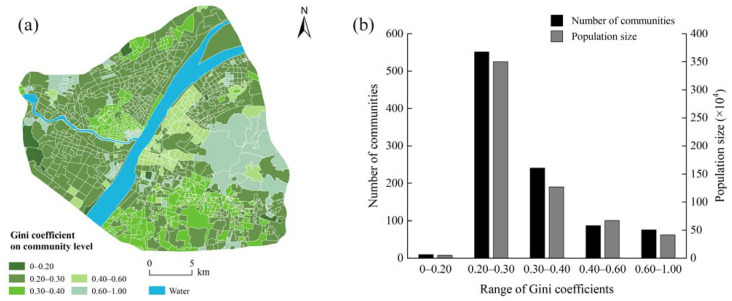
UGS equity of communities (**a**) and statistical analysis of number and population of communities (**b**).

Based on the measurement of UGS accessibility and the quantitative results of the Gini coefficient, we plotted the spatial distribution of the Gini coefficient of each community in central Wuhan (Figure 9a), and then, we further counted the number and population size of communities with different UGS equity (Figure 9b). Overall, the Gini coefficients of most communities are below 0.40, indicating that the distribution of UGS in central Wuhan is relatively fair. It is worth noting that with Yangtze River as the boundary, the left bank of the Yangtze River exhibits a better UGS equity, whereas the right bank displays a worse one. The reason for this may lie in the fact that there are many small but numerous parks scattered along the left bank of the Yangtze River, and these spatially laid out well micro-miniature parks are essential to improving the equity of the UGS. Unlike the distribution pattern of green space accessibility, the number of communities with Gini coefficients ranging from 0.20 to 0.30 is up to 552, accounting for more than 50% of the community number in the study area, and these communities also house more than half of the population of central Wuhan. Although most communities in the study area perform well in terms of equity, there are still 163 communities (*G_comm_* > 0.40) in urgent need of equity improvement. These poorly equitable communities are spread throughout the city and cover a population of over one million. Moreover, we analyze the distribution of communities with different UGS equity in the core areas (R = 1.2 km) and non-core areas (R = N). The results show that the communities with good equity (G*_comm_* ≤ 0.40) are primarily distributed in core areas (total area = 255.10 km^2^), while they are less distributed in the non-core areas (Total area = 125.74 km^2^). The area of communities with poor equity (G*_comm_* > 0.40) is 40.62 km^2^ in the core area and 64.01 km^2^ in the non–core area.

## 4. Discussion

### 4.1. Classification of UGS from a Spatial Perspective

The classification of UGS has been confirmed to be influenced by several factors such as government planning, economics, and traffic conditions [76,77,78]. Traditionally, UGSs are classified in terms of the area of parks and some simple greening indicators. In recent years, new classification criteria have been proposed officially in China [79], such as grading all the UGSs according to green space service radius and green area: target land area ratios. However, UGS accessibility and attractiveness are not taken into consideration in these official classification criteria, thus UGS equity is difficult to measure according to these criteria. In many rapidly developing Chinese cities, the transportation network is an essential driver of local economic growth and an essential indicator of how residents enjoy the basic implementation of various public services. This study confirms the imbalance between the spatial layout of green infrastructure and population distribution in Wuhan. People want a well-developed transportation network to make it easier for them to enjoy green space services. Spatial syntax is based on the mechanism of interaction between urban road networks and urban formation from the perspective of human activities. The traditional spatial syntax software is less compatible with GIS, which is not conducive to spatial analysis in complex urban scenarios. Compared with the traditional spatial syntax software, the sDNA model adds many new spatial parameters to reflect the traffic situation, which helps to better analyze UGS equity, further making more targeted policy recommendations. Therefore, this study uses the improved spatial syntax (sDNA) to explore a more objective and accurate method of UGS equity assessment in central Wuhan.

### 4.2. UGS Equity Assessment with Population Taken into Consideration

The equitable distribution of UGS is a key factor to achieve social equity and improve residents’ well-being, and an effective pathway to build a green and low-carbon city. Exploring the spatial relationship between UGS equity and population distribution can contribute to designing more effective green space planning strategies. However, most previous studies of UGS equity have been conducted from a socioeconomic perspective [80,81]. Few studies have investigated the impact of population spatial distribution on green space equity, and even fewer quantitative studies on green space equity based on community-scale population spatial distribution data are available. Considering this, the current study obtained a free population distribution data with a high-resolution of 90 m from WorldPop for UGS equity evaluation in central Wuhan. The results show that more than one million people in the study area have poor UGS accessibility and equity, and they are spread across various neighborhoods in the central and peripheral areas of the city.

### 4.3. UGS Policy Recommendations

Based on the road network data, population data, and green space data, this study conducted the UGS classification and analyzed the accessibility with the aid of sDNA, and then established a framework of UGS equity evaluation in central Wuhan according to the Gini coefficient. Based on the new green space equity evaluation results, we proposed the following recommendations for the decision makers. Firstly, the results of the kernel density analysis of sDNA indicate that the traffic intensity core areas in central Wuhan exhibit a polycentric distribution, and that the traffic network intensity ranks in the top list in Old Wuhan city, the railway stations, shopping centers, and the university town Hongshan District. The number of UGSs in the core areas is significant, and in the non-core areas is low. In addition, we measure the area of communities with different accessibility and equity within the core areas and non-core areas. Most communities with good accessibility and equity are located in the core area, and the communities within the non-core area have poor UGS accessibility and equity. Therefore, some new greenfield construction projects are suggested to be landed in non-core areas as much as possible, and the traffic construction in non-core areas ought to be strengthened gradually.

Second, the results of the community-level accessibility and equity analysis indicate that a large number of communities and their residents suffer from an imbalance in green space layout. Communities with poor UGS accessibility and good equity should be added with new green spaces in a balanced way in the future. For example, the government can establish a certain number of new small parks within or around the community. Communities with good accessibility and less equity face an imbalance in resources among their residents. For example, the park distribution is relatively concentrated for some communities with large area and large population, or the disproportionateness between number of people and the distribution of green spaces greatly damages equity. Thus, matching green space resources with population size is a key strategy for the future planning of these communities, which can be successfully achieved by increasing the TNS and the amount of UGS around high population density areas. Overall, Wuhan government needs to develop different planning strategies based on the current situation of different communities in order to fully promote the construction of a green city in central Wuhan.

Finally, relevant studies of European and American cities have shown that racial and minority groups are key factors triggering differences in UGS allocations [32,82,83,84], whereas green space allocations in China are more influenced by specific factors such as natural endowments and major strategic planning. In addition to involving strategy and policy makers, UGS strategic planning requires public participation and monitoring since community residents are the direct beneficiaries of green spaces. Thus, it is highly suggestive to establish a community resident-participated UGS equity evaluation mechanism [85]. Therefore, the future greening strategy of central Wuhan should follow more of a “bottom-up” planning concept and fully incorporate the suggestions from different communities and residents.

### 4.4. Limitations and Future Research

There are some limitations in this study. First, green spaces less than 1 hm^2^ in area were excluded in our data selection, and some pocket parks, private gardens, and small greenways were ruled out. Second, due to the limited data, we only considered the population size, but the social attributes of the population such as gender, age distribution, income level, and education level were not taken into the UGS equity assessment framework. Considering this, future studies are suggested to explore the impact of micro and small green spaces on residents’ access to green space services. In addition, the comparative analyses of UGS accessed by the groups with different socioeconomic statuses and social attributes are suggested to be taken into the UGS equity assessment framework so as to enhance the validity and relevance of policy recommendations.

## 5. Conclusions

Based on traffic road network data, UGS information data, and population distribution data, this study quantifies the UGS equity of 965 communities in central Wuhan with the aid of an sDNA model and the Gini coefficient. The results show that there are significant differences in UGS equity among different communities, and that the UGS equity of some communities with good UGS accessibility remains to be further improved. Communities with poor accessibility and equity are spread across the central and peripheral areas of the city. We further propose future greening recommendations at the city level and community level based on the strength of the traffic network and the current status of green spaces in each community. This study is of theoretical significance for constructing community living circles and green city, and future research is suggested to further explore the equity assessment of small and micro green spaces. In order to propose more objective and reasonable greening strategies, community residents’ social attributes and economical status should be included in the framework of UGS equity evaluation.

## Figures and Tables

**Table 1:** Number of UGSs in core and non-core areas.

Scale	Core Area	Non-Core Area
City-scale (R = N)	188	118
Local-scale (R = 1.2 km)	203	103

## Data Availability

Not applicable.
